# Zinc Chloride Smoke Inhalation Induced Severe Acute Respiratory Distress Syndrome: First Survival in the United States with Extended Duration (Five Weeks) Therapy with High Dose Corticosteroids in Combination with Lung Protective Ventilation

**DOI:** 10.1155/2017/7952782

**Published:** 2017-07-26

**Authors:** Hafiz Mahboob, Robert Richeson III, Robert McCain

**Affiliations:** ^1^University of Nevada School of Medicine, Reno, NV, USA; ^2^Renown Regional Medical Center, Reno, NV, USA; ^3^U.S. Department of Veteran Affairs, Ioannis A. Lougaris Veteran Affairs Medical Center, Reno, NV, USA

## Abstract

Zinc chloride smoke bomb exposure is frequently seen in military drills, combat exercises, metal industry works, and disaster simulations. Smoke exposure presents with variety of pulmonary damage based on the intensity of the exposure. Smoke induced severe acute respiratory distress syndrome (ARDS) is often fatal and there are no standard treatment guidelines. We report the first survival of smoke induced severe ARDS in the United States (US) with prolonged use of high dose steroids (five weeks) and lung protective ventilation alone. Previously reported surviving patients in China and Taiwan required extracorporeal membrane oxygenation (ECMO) and other invasive modalities. We suggest that an extended course of high dose corticosteroids should be considered for the treatment of smoke inhalation related ARDS and should be introduced as early as possible to minimize the morbidity and mortality. We further suggest that patients with smoke inhalation should be observed in the hospital for at least 48 to 72 hours before discharge, as ARDS can have a delayed onset. Being vigilant for infectious complications is important due to prolonged steroid treatment regimen. Patients must also be monitored for critical illness polyneuromyopathy. Additionally, upper airway injury should be suspected and early evaluation by otorhinolaryngology may be beneficial.

## 1. Introduction

Zinc chloride smoke bomb inhalation can induce a severe ARDS which is a lethal condition with an extremely high mortality rate. Two cases of smoke bomb induced severe ARDS survival have been previously reported in China and Taiwan [1, 2]. In the United States (US), a single case series of smoke bomb related ARDS reported two soldiers who succumbed to severe ARDS [3]. No smoke bomb induced severe ARDS survival has been reported in the US, to the best of our literature review. We successfully treated this patient with five weeks of high dose corticosteroids and did not use ECMO or other modalities, required by previously reported surviving patients in China and Taiwan. This case suggests that high doses of corticosteroids for an extended period might be beneficial for treatment of smoke bomb induced ARDS along with protective ventilation.

## 2. Case Report

A 32-year-old white male was admitted to our facility seventy-two hours after inhalation of emissions from a smoke bomb with very severe hypoxemia with an oxygen index of 58. The exposure was from a smoke bomb 1A, which generates 4000 cubic feet of zinc chloride smoke within thirty seconds. The victim was playing the role of a mock patient during a mining industry drill and was exposed to smoke in an enclosed mine analog (container) for approximately ten to fifteen minutes without any protective equipment. He developed shortness of breath and throat and chest discomfort immediately after the exposure and was provided oxygen at site. He then reported to a local emergency room, within one hour of the exposure. On initial presentation, he was tachypneic with a respiratory rate (RR) of 24 per minute and had mild hypoxia with an oxygen saturation of 87% on room air (Table 1). The State Poison Control Center confirmed that the main ingredients of the smoke were zinc chloride and carbon monoxide. He received treatment with bronchodilators (albuterol-ipratropium by nebulization), oxygen, and one dose of intravenous (IV) methylprednisolone 125 mg. His carboxyhemoglobin level was within normal limits. He was discharged home from the emergency department with a five-day course of oral prednisone 60 mg daily and an albuterol inhaler.

**Figure 1:**
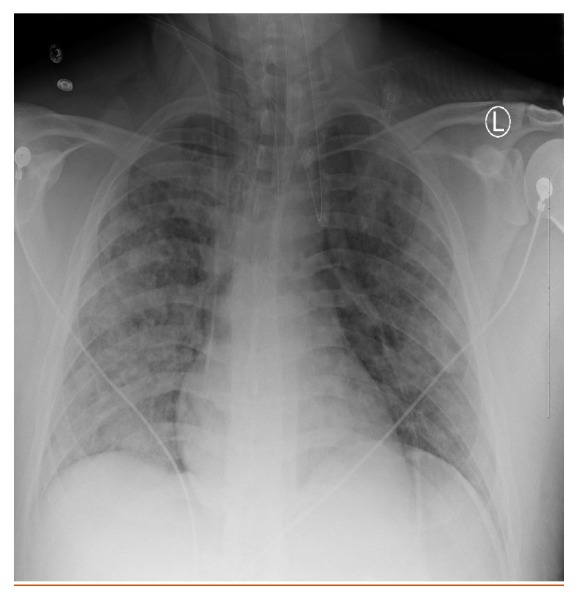
Chest X-ray.

**Figure 2:**
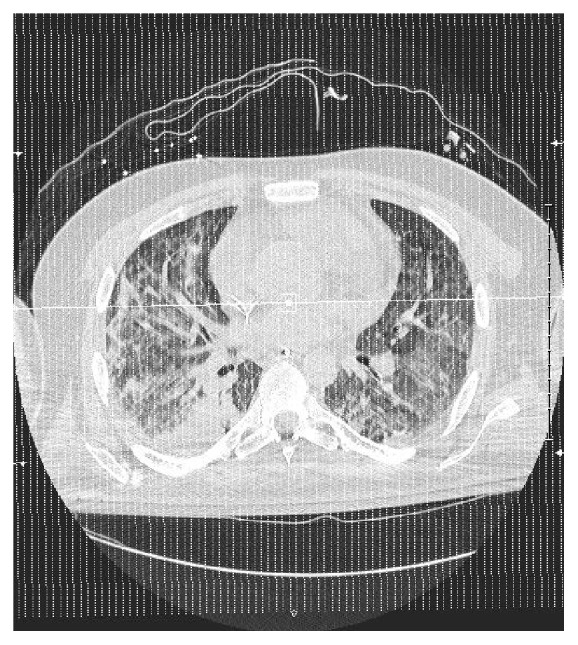
Chest CT scan.

Three days later, he presented to an outside facility for worsening shortness of breath and cough. He had profound hypoxia with an oxygen saturation of 34% on room air, improving only to 58% on 100% oxygen. His pulse was noted to be 170 per minute, and his RR was 50 per minute. At this point, he required intubation due to severe acute hypoxemic respiratory failure. His arterial blood gases (ABG) showed pH of 7.39, pCO2 of 26, and pO2 of 35 on 100% oxygen. His clinical presentation and lab findings are shown in Table 1. Electrocardiogram revealed sinus tachycardia with right axis deviation and nonspecific ST and T-wave changes. Chest X-ray (Figure 1) and chest computed tomography (CT) scan (Figure 2) showed increased opacities in the perihilar region in upper lobes bilaterally with relative sparing of the lower lobes, consistent with ARDS. No lobar consolidation was visible on chest imaging. Patient had normal left ventricular function on echocardiogram. He was placed on mechanical ventilation on volume control mode with PEEP of 20, RR of 20, and tidal volume (TV) of 8 cc/kg and was transferred to our facility.

He was admitted to the intensive care unit (ICU) and treatment was continued with high dose intravenous corticosteroids and lung protective ventilation strategy with low TV 6 cc/kg, PEEP of 18, and peak inspiratory pressure (PIP) of 28 and he was started on inhaled epoprostenol at a rate of 360 mcg/hour. Please refer to the table for complete steroids regimen (Table 2). Piperacillin/tazobactam and vancomycin were administered intravenously for ten days to cover empirically for ventilator associated pneumonia.

After completion of ten days of intravenous antibiotics and initial course of tapering intravenous steroids, he had persistent systemic inflammatory response syndrome (SIRS) criteria, and there was no change in his chest X-ray or ventilator mechanics and his oxygen index was still in the severe range (100–150) but improved from admission oxygen index of 58. All cultures were negative including follow-up bronchoalveolar lavage (BAL) cultures obtained on day eight and day eleven. He was not fluid overloaded clinically and left ventricular function was normal by echocardiogram, although he had mild pulmonary hypertension with an estimated right ventricular systolic pressure (RVSP) of 40. Nonspecific inflammatory markers were extremely elevated with an erythrocyte sedimentation rate (ESR) of greater than 120 and C-reactive protein (CRP) level of 30.97 (Table 1). Infectious disease consultant agreed that an infectious etiology was unlikely for his ongoing fever and other positive SIRS criteria. The decision was made to resume high dose intravenous steroids on hospital day 13 (Table 2).

On the third week of mechanical ventilation, three to four days after resuming high dose IV steroids, his fraction of inspired oxygen (FiO2) and PEEP along with his inhaled epoprostenol was successfully weaned to levels safe enough to perform elective tracheostomy.

On day sixteen of hospitalization, percutaneous tracheostomy was performed. The proximal trachea revealed minimally inflamed thick whitish eschar/debris and this was partially removed and sent to the laboratory for evaluation and ultimately revealed necrotic debris with fungal elements consistent with aspergillus. CT scan of the head and neck did not reveal invasive disease. Ear nose and throat (ENT) surgeon was consulted and upper airway evaluation and debridement were performed in the operating room on day twenty but only superficial debris was removed from the palate.

While his blood and initial BAL cultures did not show fungal growth, his cultures from tissue biopsy/eschar-debris from the debridement site and later BAL specimens grew aspergillus. He was also found to be positive for serum Beta-D glucan and galactomannan. On day 22, per infectious disease recommendations, he was then empirically initiated on voriconazole for mucosal/mucocutaneous bronchopulmonary aspergillosis. He continued this regimen for a total of 28 days, along with ongoing steroids for chemical pneumonitis/ARDS secondary to smoke. It should be noted that his chest X-ray and oxygenation were already showing some improvement the week before antifungal therapy was started.

Lung protective mechanical ventilation was continued, using PCMV-APV mode, high PEEP (max 20) with inhaled epoprostenol, low TV (6 cc/kg), PIP of 28 initially, and an initial paralysis for first four days using atracurium. Epoprostenol was used as continuous inhalation at dose of 0.05 mcg/kg/min at 12 cc/hour (360 mcg/hour) and was later tapered off over forty-eight hours. He was on epoprostenol inhalation for a total of twelve days. Proning therapy was considered but not employed.

After spending twenty-three days in ICU, he was transferred to a long-term acute care (LTAC) facility for his ongoing need of mechanical ventilation. Steroid therapy was held for one week and then was restarted with oral prednisone for two more weeks. (Last dose of steroids was at end of week 6 of smoke exposure.) He remained in LTAC facility for an additional period of five weeks. Weaning trials with intermittent pressure support and continuous positive airway pressure (CPAP) were initiated from ventilation day thirty and eventually he was liberated from mechanical ventilation. He was on mechanical ventilation for a total period of fifty days.

Of note, his hospital course was further complicated by a left subclavian vein thrombosis, and acute tubular necrosis, later requiring intermittent hemodialysis. The patient recovered completely from his acute tubular necrosis and dialysis was later terminated. He was then discharged to rehabilitation center from LTAC facility without any respiratory complaints and off oxygen. He spent four months in rehabilitation center primarily for critical illness polyneuromyopathy and was then discharged home.

He is now ambulating well without any clinical evidence of dyspnea or oxygen requirements.

## 3. Discussion

Smoke bombs are commonly used for leak detection in mining, the metal industry, fire simulation exercises, disaster drills, and the military for drills and combat exercises [4–6]. The most commonly used mixture for smoke bombs contains zinc oxide and a chlorine donor, which leads to the formation of fine particles of zinc chloride producing a grey-white smoke [4]. The first case of zinc chloride smoke bomb inhalation injury was reported in 1945 by Evans during World War II, resulting in the demise of ten soldiers [7].

Smoke bomb inhalation is associated with a variety of airway and lung injuries based on the duration and concentration of smoke exposure. Schenker and colleagues described an incident of zinc chloride smoke exposure because of detonation of a smoke bomb in an airport disaster drill. Exposed participants presented with upper respiratory tract symptoms. Frequency of occurrence of the symptoms and severity of the symptoms correlated with proximity to the site and duration of the exposure. In this incident, the exposure was not intense and symptoms resolved over several days without any permanent functional decline [5].

However, sometimes even a modest exposure to zinc chloride smoke can cause significant late and long-term decline in lung functions despite mild initial symptoms. A case series of thirteen soldiers exposed to zinc chloride smoke during a combat exercise showed a statistically significant decline in their lung diffusion capacity and total lung capacity of 16.2% and 4.3%, respectively, four weeks following the exposure. These findings correlated with elevated plasma zinc levels in all the patients. Lung function and volumes normalized after six to twelve months' postexposure. These patients experienced ongoing dyspnea on exertion despite normalization of lung function and volumes [8].

Intense and prolonged exposure to zinc chloride smoke has been found to cause severe lung injury and severe ARDS with high mortality [3, 6, 7]. The onset and severity of smoke bomb inhalation related lung injury depend on the concentration of the inhaled smoke and duration of exposure. There is one reported case where a patient was exposed to smoke for only 10–15 minutes without wearing a protective breathing apparatus and developed severe ARDS within 48 hours of exposure [1]. In another case series, victims were exposed to hexachloroethane (a chlorine donor for zinc chloride smoke) smoke for only 1-2 minutes without wearing gas masks and developed severe ARDS over the course of two weeks [9].

Our patient was not wearing a mask and was exposed to smoke in a contained space. Symptoms began immediately after the exposure and he was treated with oral prednisone 60 mg as an outpatient, after initial methylprednisolone bolus of 125 mg. Unfortunately, he returned seventy-two hours later with severe ARDS. His second presentation was likely delayed due to outpatient use of steroids.

Zinc chloride smoke bomb exposure is also associated with variety of other pulmonary and airway complications including pneumomediastinum, pneumothorax, and emphysematous bullae [1, 10, 11]. Matarese and Matthews reported another case of zinc chloride inhalation in US, where a patient developed severe emphysematous blebs after smoke inhalation which was complicated by pneumothorax. This patient survived with gradual recovery occurring over several months but chest X-ray remained abnormal with emphysematous blebs [10].

Zinc smoke exposure has also been reported to cause vascular lesions including endothelial cell proliferation and extensive interstitial and intra-alveolar space fibrosis [3, 9, 12]. Milliken with his colleagues reported a case where patient succumbed to acute severe interstitial pulmonary fibrosis after zinc chloride smoke bomb exposure [12].

This patient had a significant upper airway and palatal burn, which required debridement by an ENT surgeon. These burns were complicated by the mucocutaneous aspergillus infection and this complication was likely secondary to steroid and antibiotic treatments. Being on prolonged steroid treatment regimen, it is important to remain vigilant for any infectious complication as well as seeking an ENT evaluation for upper airway injury early on.

Zinc chloride smoke bomb induced severe ARDS has an extremely high mortality rate [3, 6, 7] and only two cases of survival [1, 2] have been reported. However, no smoke bomb induced severe ARDS survival has been reported in the US, to the best of our literature review. There are no standard guidelines available for the treatment of acute respiratory distress syndrome secondary to inhalation of smoke from a smoke bomb.

Traditionally lung protective ventilation strategy and corticosteroids have been the mainstay of treatment for smoke inhalation induced lung injury and both therapies have shown survival benefits [1, 2, 9, 13–15]. Amato and colleagues showed that use of a lung protective ventilatory strategy with low TV, high PEEP, and low plateau pressure of less than 30 cm H_2_O increased survival rate up to 62% compared with 29% when conventional ventilation was used [13].

Other pharmacological treatment modalities such as N-acetylcysteine, L-3,4-dihydropyridine, methylene blue, inhaled nitric oxide [2, 9], exogenous surfactants [9], and penicillamine [16] have also been tried in individual cases of smoke exposure induced lung injury. Intravenous and nebulized N-acetylcysteine is thought to increase the urinary excretion of zinc. L-3,4-Dihydropyridine has been proposed to arrest collagen deposition [2] and intravenous methylene blue is used to treat methemoglobinemia and for respiratory support [9]. However, none of these treatment modalities have shown any survival benefits [9]. In addition to pharmacological modalities, other invasive strategies such as videothoracoscopic excision of emphysema bullae and recurrent chemical pleurodesis have been tried for the associated complications of pneumothorax and pleural effusions, respectively [2].

We used epoprostenol as rescue therapy in our patient. Nitric oxide inhalation has shown improved oxygenation for a short period of 48 hours in cases of severe ARDS but it did not show any survival benefits [2, 13, 15, 17]. There are no available strong randomized clinical studies for the effectiveness of inhaled prostaglandins in the treatment of ARDS. One clinical trial, which included 14 critically ill children with ARDS, did not show survival benefits of prostacyclin versus placebo [18]. However, observational studies and case series have shown efficacy of inhaled prostaglandins for improved oxygenation in severe hypoxemia due to ARDS. A recent retrospective chart review, which included 16 patients treated with inhaled epoprostenol, showed that epoprostenol improved oxygenation (defined as an improvement in P_aO2_/F_IO2_ of >10% from the baseline) in 62.5% of the treated patients [19, 20]. Therefore, inhaled prostaglandins have been increasingly used lately as a rescue therapy for severe refractory hypoxemia in severe ARDS given its cost effectiveness compared to inhaled nitric oxide as well as similar efficacy for improving oxygenation and safety outcomes [21].

There are no prospective trials available on the utility of prone positioning in cases of smoke inhalation induced ARDS. One case report of smoke induced severe ARDS did not show any survival benefit of proning therapy [2]. However, one recent prospective RCT study (PROSEVA trial) has shown mortality benefit for early introduction of prone positioning (implied early within 36 hours, for 18–20 hours daily) as an adjunct therapy with protective lung mechanical ventilation for the treatment of severe ARDS [22]. Early prone positioning leads to effective alveoli recruitment during the acute exudative phase of ARDS, thus improving oxygenation by minimizing the shunt in patients with severe refractory hypoxemia [23]. We started high dose steroids with high PEEP and inhaled epoprostenol as rescue therapy for severe hypoxemia which helped us to wean his FiO2 gradually and improve oxygen index. We would certainly have tried prone positioning if initial rescue modes with epoprostenol, high PEEP, and high dose steroids would have failed. We urge to utilize proning as early adjunct therapy in cases of smoke exposure induced severe ARDS early on and/or in cases of severe refractory hypoxemia despite use of other rescue modalities when there is no contraindication to proning.

ECMO use is not well studied in cases of smoke induced severe ARDS in perspective trials. However, there have been reported cases of severe ARDS due to smoke inhalation who survived with the use of ECMO [1]. Bartlett et al. reported that, among adult patients with ARDS who were treated with ECMO, the survival rate was 61% [24]. ECMO is now being increasingly utilized as a rescue therapy for severe hypoxemia in cases of severe ARDS when there is no absolute or relative contraindication for its use. A recent trial showed a reduction in mortality and severe disability rates at six months following the use of ECMO [25]. Extracorporeal life support organization (ELSO) guidelines suggest considering using ECMO in cases of severe ARDS in adults when there is PaO_2_/F_I_O_2_ ratio of 70–80 mmHg, Murray score > 3, and pH < 7.2 [26–28]. Absolute contraindications to the use of ECMO are irreversible lung disease with no indication for lung transplantation or severe brain damage associated with major cerebral infarction or severe intracranial bleeding. Other situations such as immunosuppression, bleeding, and mechanical ventilation at high settings (F_I_O_2_ > 0.9, PIP > 30 mmHg) for >7 days are considered as relative contraindication to ECMO therapy [27–30]. For implication of ECMO therapy, a ventilation strategy with a PIP of less than 25 cmH_2_O, PEEP of 5–15 cmH_2_O, and F_I_O_2_ of 0.3 is preferred [30–32]. We are well quipped community/university teaching hospital but we are not a recognized ECMO center. We treated our patient with available rescue therapies with epoprostenol and high dose corticosteroid in conjunction with high vent settings (PEEP 20 mx, PIP 28, and FioO2 100% initially) which were weaned down gradually. We considered ECMO as second option and would have transferred him to an outside ECMO center, in case our initial rescue strategy would have failed.

The role of corticosteroids overall in the treatment of acute lung injury and ARDS has been controversial. One study conducted by Bartlett et al. suggests that high dose glucocorticoids do not decrease the frequency of lung injury in patients at risk. It also suggested that high dose glucocorticoids do not modify the disease course [24]. Other studies by Wheeler and Bernard proposed that, in addition to modifying the inflammatory response, high dose corticosteroids reduce mortality. None of these studies included patients with smoke inhalation related ARDS [33]. However, there have been cases reported in English and Asian literature where early introduction of high dose intravenous steroids gave favorable outcomes in zinc chloride smoke inhalation related chemical pneumonitis [1, 2, 9]. Ishimoto and colleagues in Japan reported two cases of inhalational exposure to zinc fumes and zinc powder while working in a boathouse, without using protective equipment. These patients presented early, were admitted to the hospital, and responded well to early administration of intravenous steroids and noninvasive positive pressure ventilation (NIPPV) with favorable clinical outcomes [34]. We did not use NIPPV because of the severity of respiratory distress and profound hypoxemia at presentation. Johnson and Stonehil presented three cases of severe, generalized chemical pneumonitis secondary to zinc chloride smoke exposure [35]. Again, all these patients presented early and responded well. This emphasizes that close, in-hospital observation with early introduction of intravenous steroids can greatly reduce the severity of respiratory failure and thus potentially shorten the duration of hospitalization and decrease the overall morbidity and mortality.

We treated the patient with a 10-day course of intravenous antibiotics to empirically cover for hospital acquired pneumonia (HAP) along with the initial course of steroids [36]. There are no comparative studies available regarding the individual advantages of methylprednisolone versus hydrocortisone for the treatment of ARDS. Both treatment choices have been used in different clinical trials. There is no recommended standard steroid treatment regimen for smoke induced ARDS in the literature. However, there is one recent RCT study which favored the use of intravenous hydrocortisone (for 7 days) over the placebo for improved lung function (duration free from mechanical ventilation, reintubation rate) in patients with ARDS secondary to sepsis. We used intravenous hydrocortisone for one week from day 7 to day 13 [37].

However, he had persistent signs of SIRS with fever and leukocytosis despite optimal empiric coverage for HAP. Microbiology and cultures were negative with extremely elevated ESR and CRP with ongoing signs of inflammation. It was at this point that we went back to a high dose IV methylprednisolone for the continued steroid treatment regimen for smoke induced severe ARDS and he significantly responded and improved over the course of the treatment. Superficial fungal infection was felt to be a minor and secondary complication, mostly in the upper airway eschar/debris secondary to the delayed initial injury, although he did receive treatment for 28 days with voriconazole as per infectious disease recommendations.

In summary, ECMO treatment in addition to steroids and protective lung ventilation has been reported beneficial in individual case reports when treating smoke bomb related ARDS. In our patient, we did not use N-acetylcysteine, L-3,4-dehydroproline, penicillamine proning, or ECMO and the patient did not require any additional invasive procedures. This patient survived with prolonged high dose steroids for five weeks and protective lung ventilation with low tidal volume, high PEEP, and inhaled epoprostenol alone.

The patient also recovered from his acute tubular necrosis. He was discharged to a rehabilitation facility off oxygen without any respiratory complaints, primarily for his critical illness polyneuromyopathy. From the rehabilitation center, he was then discharged home but still has some residual peripheral neuropathy. It should be noted that being vigilant for critical illness polyneuromyopathy is crucial in cases such as these that require prolonged steroid treatments and especially if paralytics are also used [38, 39].

## 4. Conclusion

High doses of corticosteroids for an extended period might be beneficial for treatment of smoke bomb induced severe ARDS and should be considered as a single agent pharmacologic treatment modality along with protective ventilation. These patients must be observed in the hospital for at least forty-eight to seventy-two hours before discharge, as ARDS can have a delayed onset, especially if treated with steroids early. Being vigilant for any infectious complications and critical illness polyneuromyopathy is prudent due to prolonged steroid treatment regimen. Additionally, upper airway injury should be suspected and early upper airway and proximal trachea evaluation by ENT may be beneficial. Zinc chloride smoke use must be minimized due to the morbidity and mortality associated with the smoke exposure and it should be replaced with another nontoxic or less toxic substance. Future case studies may further enlighten the treatment options for this rare, but frequently lethal condition.

## Figures and Tables

**Table 1:** Patient characteristics.

Variable	Value
*Initial encounter (day 1)*	
Pulse	97/minute
Respiratory rate	25/minute
Blood pressure	106/67 mmHg
Oxygen saturation	
Room air	87%
1-2 L oxygen	91%
*Second encounter (day 3)*	
Pulse	170/minute
Respiratory rate	50/minute
Blood pressure	87/55 mmHg
Temperature	100.5 F
*Oxygen saturation*	
Room air	48%
100% oxygen	58%
*Laboratory data *	
WBC	27.8 × 10^9^/L
Neutrophil	73%
Hemoglobin	16.2 g/dl
Hematocrit	48.8%
Platelets	76,000 × 10^9^/L
Sodium	136 mEq/L
Potassium	5.1 mEq/L
Chloride	98 mEq/L
Bicarbonate	13 mEq/L
BUN	22 mg/dl
Creatinine	2.04 mg/dl
Anion Gap	25 mEq/L
D-dimer	3410 mg/L
Troponin	0.5 ng/mL
BNP	129 pg/mL
ESR	>120 mm/hr
CRP	28.20 mg/L
*ABG on 100% oxygen*	
pH	7.39
pO2	35 mmHg
pCO2	26 mmHg

ABG: arterial blood gas.

**Table 2:** Details of corticosteroids regimen (day since exposure to smoke, drug and route of administration, frequency and strength, and total number of doses).

Day	Drug and route	Frequency/strength	Total doses
1	Methylprednisolone IV	125 mg	1

2-3	Prednisone oral	60 mg	2

3–6	Methylprednisolone IV	125 mg	1
		62.5 mg every 6 H	10
		50 mg every 8 H	3

7–12	Hydrocortisone IV	100 mg every 12 H	3
		50 mg every 12 H	14

13–21	Methylprednisolone IV	125 mg every 6 H	12
		40 mg every 6 H	12
		40 mg every 8 H	03
		40 mg every 12 H	05
		30 mg	01

28–42	Prednisone oral	40 mg daily	7
		30 mg daily	4
		20 mg daily	3

IV: intravenous, H: hours, day since exposure to smoke.
